# High-Throughput Metabolic Profiling of Diverse Green *Coffea arabica* Beans Identified Tryptophan as a Universal Discrimination Factor for Immature Beans

**DOI:** 10.1371/journal.pone.0070098

**Published:** 2013-08-01

**Authors:** Daiki Setoyama, Keiko Iwasa, Harumichi Seta, Hiroaki Shimizu, Yoshinori Fujimura, Daisuke Miura, Hiroyuki Wariishi, Chifumi Nagai, Koichi Nakahara

**Affiliations:** 1 Innovation Center for Medical Redox Navigation, Kyushu University, Fukuoka, Japan; 2 Frontier Center for Value Creation, Suntory Business Expert Limited, Osaka, Japan; 3 Hawaii Agriculture Research Center (HARC), Kunia, Hawaii, United States of America; Laurentian University, Canada

## Abstract

The maturity of green coffee beans is the most influential determinant of the quality and flavor of the resultant coffee beverage. However, the chemical compounds that can be used to discriminate the maturity of the beans remain uncharacterized. We herein analyzed four distinct stages of maturity (immature, semi-mature, mature and overripe) of nine different varieties of green *Coffea arabica* beans hand-harvested from a single experimental field in Hawaii. After developing a high-throughput experimental system for sample preparation and liquid chromatography-mass spectrometry (LC-MS) measurement, we applied metabolic profiling, integrated with chemometric techniques, to explore the relationship between the metabolome and maturity of the sample in a non-biased way. For the multivariate statistical analyses, a partial least square (PLS) regression model was successfully created, which allowed us to accurately predict the maturity of the beans based on the metabolomic information. As a result, tryptophan was identified to be the best contributor to the regression model; the relative MS intensity of tryptophan was higher in immature beans than in those after the semi-mature stages in all arabica varieties investigated, demonstrating a universal discrimination factor for diverse arabica beans. Therefore, typtophan, either alone or together with other metabolites, may be utilized for traders as an assessment standard when purchasing qualified trading green arabica bean products. Furthermore, our results suggest that the tryptophan metabolism may be tightly linked to the development of coffee cherries and/or beans.

## Introduction

Coffee is one of the most popular beverages consumed worldwide. There are two commercially important species of the genus *Coffea* in the Rubiaceae family, *C. canephora P*. and *C. arabica L.,* which occupy approximately 30% and 70% of world products, respectively [1]. Although arabica is highly vulnerable to climate changes [2] and diseases such as rust, it generates superior flavors and produces high-quality beverages. To date, many arabica varieties (or cultivars) have been established, and the breeding is ongoing with the aim of not only maintaining the genetic variation of the species, but also to improve both the yield and quality of the products [1].

For those who engage in trading/processing/consuming the green bean products, it is regarded that the highest quality commodity contains less foreign materials and/or deficient beans [3] [4] [5], and consists of only beans derived from fully matured coffee cherries. Contamination with foreign materials (*e.g.*, stones, sticks and lumps) and defective beans (*e.g.*, black beans and broken pieces) can be clearly judged by appearance, thereby allowing the removal of nearly all contaminants using various devices, such as a wind force sorters, sieve sorters, specific gravity sorters and metal detectors, in addition to hand-picking. In contrast, the contaminated immature beans are very difficult to distinguish from fully mature beans, although some, but not all, may be eliminated during the process of converting the harvested coffee cherries into the green beans (*e.g.*, wet-drying method). Since immature beans are considered to be the main cause of bad flavors in the resultant coffee beverage, it is critically important to accurately assess the quality of the green arabica beans in terms of maturity [3] [6].

To date, many methods have been used in coffee green bean analysis, including near infrared spectroscopy (NIRS)[7], visible micro-Raman spectroscopy [8], Fourier transform infrared (FTIR) spectroscopy [5], nuclear magnetic resonance (NMR)[9] and mass spectrometry (MS)[10], in an attempt to classify the species between arabica and canephora, the origins and cultivars within arabica species, or to discriminate defective from non-defective green beans. Among them, high performance liquid chromatography (LC)-MS-based metabolic profiling techniques are often used in metabolomics studies due to their high sensitivity and selectivity, and can provide a comprehensive, quantitative and unbiased (in some cases) view of wide arrays of metabolites in biological samples [11] [12] [13]. However, there has been little information concerning the metabolomic analysis of green coffee beans at different stages of maturity. Therefore, elucidating the metabolomic dynamics associated with maturity could lead to the identification of previously undescribed biomarkers that may be useful for evaluating the quality of arabica beans.

In this study, based on the development of novel high-throughput methodologies for sample preparation and LC-MS measurement, we performed metabolic profiling of diverse arabica green beans, specifically focusing on their maturity, and characterized the chemical attributes that may be utilized for discriminating the maturity (and quality) of the beans.

## Results and Discussion

### Sample Preparation of Diverse Arabica Green Coffee Beans

**Figure 1:**
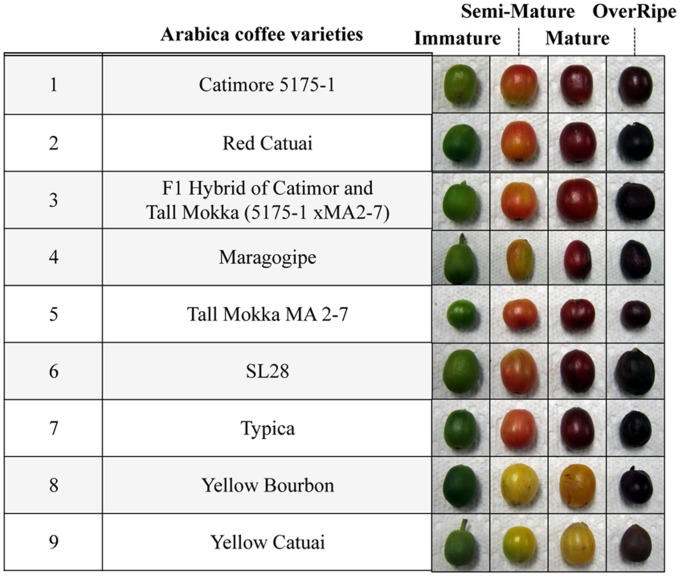
A list of the samples included in the LC-MS analysis. *Coffea arabica* cherries of nine different varieties [Catimor 5175-1, Red Catuai, F1 hybrid of Catimor and Tall Mokka (5175-1 xMA2-7), Maragogipe, Tall Mokka MA2-7, SL28, Typica, Yellow Bourbon and Yellow Catuai] and four distinct maturities (immature, semi-mature, mature and overripe) were harvested at the HARC in Hawaii.

An accurate assessment of the quality of green beans requires the identification of potent chemical compounds (or metabolite biomarkers) that can discriminate the maturity of the green beans from diverse arabica varieties. To identify such a compound, we prepared the green bean samples from cherries which were hand-harvested from nine different *Coffea arabica* varieties [Catimor 5175-1, Red Catuai, F1 hybrid of Catimor and Tall Mokka (5175-1 xMA2-7), Maragogipe, Tall Mokka MA2-7, SL28, Typica, Yellow Bourbon and Yellow Catuai] in the experimental field in Hawaii [14] [15]. Although each tree bore different developmental stages of cherries, we can easily distinguish them into four distinct groups as immature, semi-mature, mature and overripe, according to their colorful appearances (Fig. 1). Then, we harvested and processed them, converting all into the green beans by the wet-drying method. For this study, a sample set comprising a total of 108 samples (nine varieties at four developmental stages from three different trees, n = 3) was obtained.

### Development of a High-throughput Method for Sample Preparation and LC-MS Measurement

**Figure 2:**
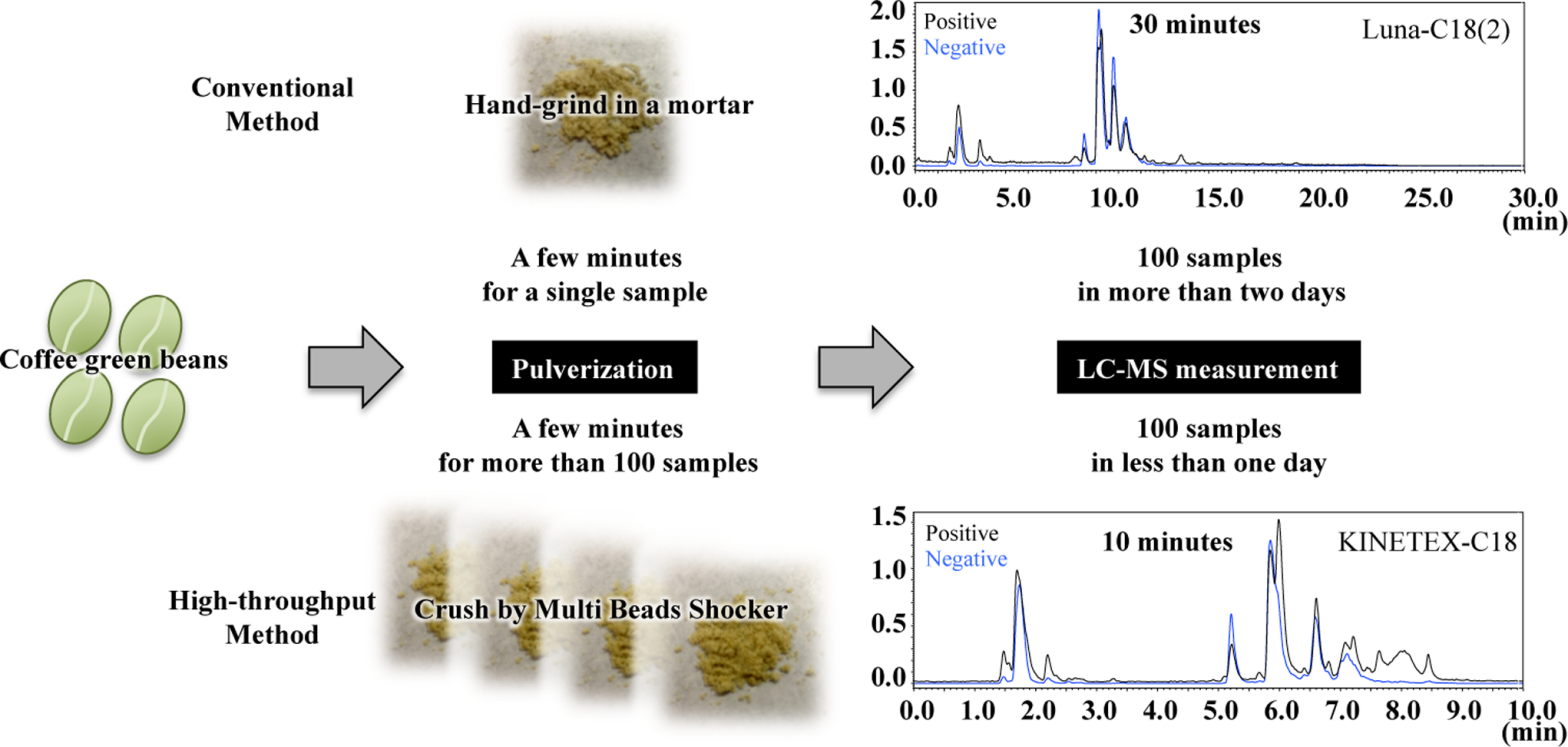
Development of a high-throughput analytical method for sample preparation and LC-MS measurement. First, green coffee beans were subjected to pulverization using a MultiBeads Shocker (Yasui Kikai, Japan), allowing us to process 18 samples in 20 seconds at once, while a conventional method using a mortar takes a few minutes for a single sample. Second, the extracted metabolites were subjected to LC-MS using a KINETEX C18 column, which allows a rapid (10 minute) separation for a single sample, which requires one-third less time than the conventional separation method.

Green coffee beans are too hard to easily crush. When preparing samples for LC-MS, the conventional method utilizing a mortar is very time- and labor-intensive. To make the sample processing easier, we used a MultiBeads Shocker (Yasui Kikai, Japan) in order to crush a lot of samples in a short time period (Figure 2). The instrument could process 18 samples simultaneously in only 30 seconds, whereas the conventional method would require more than one hour to process the same number of samples. Moreover, the mechanically crushed particle size appeared to be almost equal to that obtained using a mortar. Thus, a very high-through put method for sample preparation has been established, which might be useful for processing other hard materials that are difficult to crush, such as the seeds of fruits.

The LC-MS measurement itself is another time-consuming step. After the extraction of metabolites from the crushed powder, the conventional reverse-phase (RP) LC-MS measurement takes approximately 30 minutes for each sample. When there are a large number of samples to evaluate, the time required for the analysis is increased, which leads to decreased MS sensitivity and increased errors. To avoid these drawbacks, we developed a fast and efficient LC-MS analysis using a KINETEX C18 column, and successfully optimized the time program, taking only 10 minutes per sample (Figure 2). Indeed, the total ion chromatogram obtained using the conventional RP column from both positive and negative ion modes appeared to be compressed in the new 10 minute time program without altering the form of the chromatogram or losing MS sensitivity. Altogether, our new methods have largely overcome the two most time-consuming steps, sample preparation and LC-MS measurement, thereby facilitating the high-throughput analysis of coffee green beans.

### Multivariate Statistical Modeling for the Identification of a Maturity Discrimination Factor

**Figure 3:**
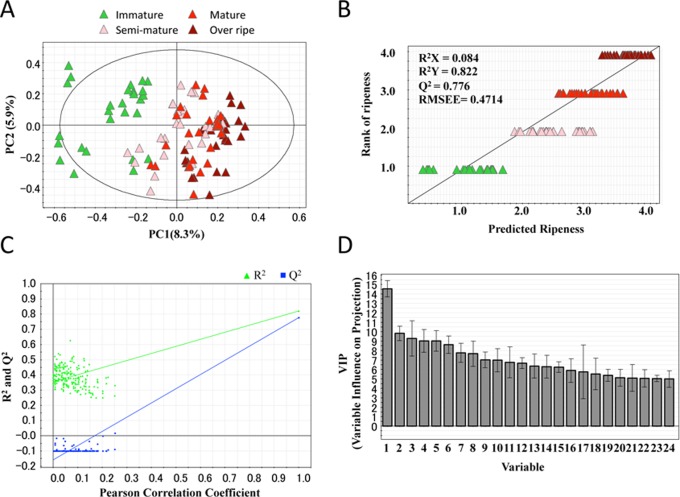
Multivariate statistical analyses. (A) The principal component analysis (PCA) score plot showed that the first two components (PC1/PC2) represented 14.2% of the variation among all samples. Each sample is colored according to the maturity in green (immature), pink (semi-mature), red (mature) or umber (overripe). The ellipse represents Hotelling’s T2, with 95% confidence in the score plots. (B) The partial least squares (PLS) regression model. The relationship between the four maturities as the rank-ripeness [immature (1), semi-mature (2), mature (3) and overripe (4)] and the 117 samples was modeled by the PLS method. One PLS component described 8.4% (R^2^X) of the variation among all samples. The goodness of fit value, R^2^Y, and the goodness of prediction value, Q^2^Y (cross-validated R^2^Y), were 0.822 and 0.776, respectively. The root-mean-square of the error of estimation (RMSEE) was 0.4714. (C) The validation plot (after 200 permutations) of the one-dimensional PLS model. The Y-axis represents R^2^Y (triangle in green) and Q^2^Y (squares in blue) for every model, and the X-axis designates the Pearson correlation coefficient between the original and permutated rank-ripeness. (D) The VIP plot of the PLS model. The top 24 important variables are shown according to their VIP values.

Based on the above methodology, our 108 green bean samples were subjected to LC-MS measurement. Then, the obtained LC-MS spectra were processed for peak picking and alignment, from which we obtained a total of 3,297 valid peaks. Due to the substantial amount of data obtained in LC-MS measurement, it was also necessary to employ the aid of chemometric approaches [16]. We applied two multivariate statistical techniques commonly used in metabolomics studies to isolate the most characteristic metabolite markers in our experiment. Using the SIMCA-P+ software program, a principal component analysis (PCA) was first performed in a non-biased way to simply visualize the differences in the metabolite profiles corresponding to each maturation stage. In the PCA score plot (Fig. 3*A*), where the first two principal components (PC1 and PC2) accounted for 14.2% of the original variance, it appeared that a small group of metabolite (less than 10%) were changed based on the maturity of the beans (along with the PC1-axis).

Second, to further focus on the maturity-related metabolites, we created a maturity prediction model based on the partial least squares (PLS) regression analysis [16]. For the regression (Fig. 3*B*), the 108 samples were postulated to belong to their rank corresponding to their maturity as immature (1), semi-mature (2), mature (3) and overripe (4), then we isolated the potent metabolites that best contributed to the regression model. Indeed, we were able to detect that only 8.4% of the variation correlated with the first latent variable. Moreover, the quality of the regression model, as verified, in a part, by the values of the correlation coefficient (R2, 0.822), the cross-validated correlation coefficient (Q2, 0.776) and the root mean squared error of estimation (RMSEE, 0.4714), appeared to be a good predictive ability for the model [17].

To further ensure the statistical significance of our PLS model, we performed a validation analysis in SIMCA-P, where the values for R2 and Q2 were assessed when permutated with the maturity (y-variable). If our PLS model were overfitted, the two values would not virtually change along with the X-axis describing the correlation coefficients between the permutated and original y-variable. However, the analysis showed that the R2Y-intercept was between 0,3–0.4 and the Q2Y-intercept was below zero, indicating that there was a significant decline (change) of the two values (Fig. 3*C*). Therefore, these results, together with the values of R2Y, Q2Y and RMSEE, strongly supported that our PLS regression model has substantial predictive power, with statistical significance [17]. Based on this finding, the interpreted PLS regression model was given by the VIP parameter (variables influence on projection). Using all of this information and the newly established methods, we obtained a list of metabolite peaks ranked according to the VIP values (Fig. 3*D*). As higher ranked variables (metabolite peaks) are suggested to substantially contribute to the regression, the top ranked peaks were therefore considered to be most strongly correlated with the maturity of the green coffee beans.

### Tryptophan is a Specific Marker of Immaturity in divers *Coffea arabica* Green Beans

**Figure 4:**
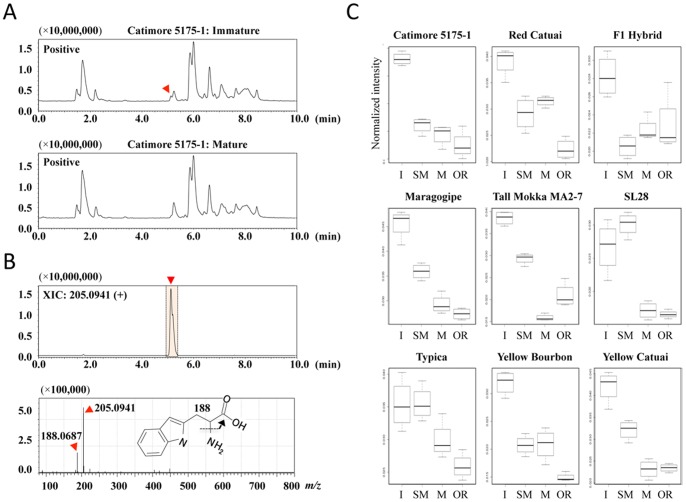
Tryptophan is a specific marker of diverse immature *Coffea arabica* green beans. (A) The total ion chromatograms derived from the LC-MS analyses of either the catimore 5175-1 immature or mature bean extract. The red arrowhead indicates a characteristic peak that specifically appeared in the green bean sample. (B) The extracted ion chromatogram (XIC) of 205.0941 (+) retained at 5.00–5.40 minutes and the mass spectrum are shown. (C) Box-and-whisker plots derived from the tryptophan in diverse varieties. The mass intensities (Y-axis) were normalized to the sum total ion counts obtained from each sample. The developmental stages (X-axis) were abbreviated as I (immature), SM (semi-mature), M (mature) and OR (overripe).

When we compared the total positive ion chromatograms between the immature green samples and those of the other developmental stages, apparent dissimilarity was observed around five minutes (Fig. 4A, arrowhead in red), although the overall forms were closely similar. Indeed, the extracted ion chromatogram (XIC) of the two top ranked peaks (m/z values, 205.0941 and 188.0687) were found to correspond to the dissimilarity (Fig. 4B, upper panel). Most interestingly, these peaks were successfully assigned to tryptophan (205.0941) and deaminated tryptophan (188.0687), respectively, based on the matched m/z values and the retention time of the standard compound (Fig. 4B, lower panel). Furthermore, the relative ion intensities of tryptophan (and deaminated tryptophan) were found to decline in close association with the maturing stages of all nine arabica varieties (Fig. 4C). Therefore, the results clearly indicate that tryptophan is a universal discrimination factor for the maturity of arabica species.

In various food products, bioactive amines, including polyamines (*e.g.* putrescine, spermidine and spermine) and biogenic amines (*e.g.* histamine, tryptamine and tyramine), are suggested to serve as important indicators of their quality [18]. Given this and our findings, it is inferred that tryptophan, either alone or together with other metabolites (e.g. amines), can be utilized as an assessment standard for the quality of green coffee beans. When it comes to practical use, however, many issues still need to be addressed. For instance, it remains to be determined whether it is applicable to those beans that were prepared from other processing methods such as the air-drying method (which are often employed in Brazil), the accuracy of the marker must be evaluated, and to what degree tryptophan alone can discriminate the immature beans in a mixture where mature beans are dominant, will thus need to be examined further.

In most plants, tryptophan plays critical roles in the growth and development of the leaves, fruit and seeds, and is involved in the biosynthesis of plant hormones such as auxin or other indole-derived compounds [19] [20]. It could be thus plausible that the relatively abundant tryptophan present in immature beans may be destined to be converted into auxin (or the derivatives) during the maturation process. If so, then the tryptophan-derived compounds may also be determined to be maturity-discrimination factors, thereby higher ranked in the VIP list obtained from our PLS regression (maturity predicting) model. Therefore, future studies, including the identity of un-annotated MS peaks in our studies, are needed to verify this hypothesis regarding the relationship between tryptophan metabolism and the maturity of coffee cherries and/or beans.

### Conclusions

This study aimed to investigate the maturity-related biomarkers in arabica green coffee beans employing non-targeted LC-MS-based metabolic profiling techniques. For this purpose, green bean samples were hand-prepared from diverse arabica varieties in Hawaii. In addition, two improved methodologies that enable for high-throughput sample preparation and LC-MS measurement were developed. As a result, we successfully identified tryptophan as being one of the specific markers of immaturity in diverse Coffea Aarabica green beans. Our finding could therefore be useful for evaluating the quality of arabica bean products.

## Materials and Methods

### Reagents used for this Study

LC-MS grade ultrapure water, acetonitrile, methanol and formic acid were purchased from Wako (Tokyo, Japan).

### Plant Material

All of the trees were planted in 1997–2000, and maintained in the fields at the Kunia experimental station of Hawaii Agriculture Research Center (HARC) on Oahu, Hawaii. Nine coffee varieties (*Coffea Arabica L.*) used for this study included Catimor 5175-1, Red Catuai, F1 hybrid of Catimor and Tall Mokka (5175-1 xMA2-7), Maragogipe, Tall Mokka MA2-7, SL28, Typica, Yellow Bourbon and Yellow Catuai [15].

### Preparation of Green Coffee Beans

Approximately ten grams of arabica cherries (fruits) of different nine varieties and four distinct maturities (immature and semi-mature, mature and overripe) were harvested by hand picking. Harvested cherries were processed within 3 hours by the wet-drying method; pulps were removed manually from the cherries and soaked in water overnight at ambient temperature (24–26°C), and then, samples were washed and air dried for 7–10 days until the final water content reached 12%. The parchment beans were threshed to remove the parchment (seed coat), resulting in green coffee beans that were ready to be traded, roasted or analyzed.

### Metabolite Extraction for the LC-MS Analysis

Conventionally, approximately two grams of green coffee beans frozen in liquid nitrogen were subjected to manual grinding in a mortar, with several minutes required to process a single sample. Alternatively, using our method, an equal amount of frozen beans was mixed with a small metal device and crushed by shaking with a MultiBeads Shocker (Yasui Kikai, Japan) at 3,000 rpm for 20 seconds. This improved method allowed us to process 18 samples at once. The coffee metabolites were then extracted using 50 mg of the crushed powder incubated with 1.5 ml of 70% methanol for 1 hour, with vortex mixing every 15 minutes. After centrifugation at 15,000 rpm for five minutes at 4°C, the supernatants were collected, diluted four times with 50% methanol and filtered using a Microcon YM-3 membrane centrifugal filter unit (Millipore, Billerica, MA). The flow-through fractions (< 3,000 Da) were subjected to a liquid chromatography-mass spectrometry (LC-MS) analysis.

### High-throughput LC-MS Measurement

The coffee extracts were separated using high-performance LC (HPLC) on either a Luna-C18 (2) column (250 × 1.0 mm, 5 μm particle size, Phenomenex, CA) or a KINETEX C18 column (250 × 2.1 mm, 1.7 μm particle size, Phenomenex, CA) coupled with electrospray ionization-time of flight (ESI-TOF)-MS using a LCMS-IT-TOF instrument (Shimadzu, Japan). The mobile phase consisted of solvent A (0.1 % formic acid) and solvent B (100 % acetonitrile), and the column oven temperature was 40°C. The gradient elution program for the Luna C18 column was as follows: a flow rate of 0.1 mL/min: 0–2 min, 5% B; 2–7.5 min, 5–55 % B; 7.5–17 min, 55–100% B; 17–23 min, 100% B; 23–24 min, 100–5% B; and was maintained at 5% B until 30 min had passed. For the KINETEX-C18 column, the conditions were as follows: the flow rate was 0.2 mL/min: 0–1 min, 5%B; 1–4.5 min, 5–25 %B; 4–5 min, 25–100 % B; 5–5.5 min, 100% B; 5.5–6.0 min, 100–5% B; and was maintained at 5% B until 10 min had passed. For ESI, the ionization parameters were as follows: ionization mode, positive and negative; mass range, *m/z* 80–800; probe voltage, +4.5 kV (positive), –3.5 kV (negative); nebulizer gas flow rate, 1.5 L/min; CDL temperature, 250 °C; heat block temperature, 250°C.

### LC-MS Data Analysis

The mass spectra obtained in the LC-MS analysis were processed for peak picking and alignment using the Profiling Solution software program (Shimadzu, Japan). All *m/z* peaks in either positive or negative ion mode were normalized to the sum total ion counts of each sample. The peaks were screened according to two criteria: at least one group, of a total of 36 categorical groups (nine varieties at four stages of maturity, n = 3) exhibited < 25% missing values and RSD values of <75%, resulting in 3,297 valid peaks.

### Multivariate Statistical Analysis

The multivariate data sets were centered, scaled to Pareto and then subjected to a multivariate statistical analysis using the SIMCA P+ ver. 12.0 software program (Umetrics, Sweden). To obtain an overview of the coffee metabolomic data, an unsupervised method, principal component analysis (PCA), was performed according to the user’s manual. On the other hand, a partial least squares (PLS; also known as projection to latent structure) regression model was created to isolate potent metabolite markers for predicting the maturity of green coffee beans. For the regression analysis, we assigned a rank number to each maturity of the samples as follows; green (1), semi-mature (2), mature (3) and overripe (4), and used these as the y-variable.

### Box-and Whisker Plots

Box-and-whisker plots were generated using the R statistical software program (http://cran.at.r-project.org/). The center line in the box denotes the median, and the bottom and top boundaries of the box denote the 25^th^ and 75^th^ percentiles, respectively. The lower and upper whiskers denote the range of the data. The Y-axis indicates the normalized peak intensity.
